# Developing an *in situ* LED irradiation system for small-angle X-ray scattering at B21, Diamond Light Source

**DOI:** 10.1107/S1600577524003205

**Published:** 2024-05-31

**Authors:** Beatrice E. Jones, Ann Fitzpatrick, Kieran Fowell, Charlotte J. C. Edwards-Gayle, Nikul Khunti, Katsuaki Inoue, Steven Daniels, Eugene Williams, Camille Blayo, Rachel C. Evans, Nathan Cowieson

**Affiliations:** a Diamond Light Source, Harwell Science and Innovation Campus, Didcot, OxfordshireOX11 0DE, United Kingdom; b Department of Materials Science and Metallurgy University of Cambridge 27 Charles Babbage Road CambridgeCB3 0FS United Kingdom; c School of Chemistry Trinity College Dublin, University of Dublin College Green Dublin Ireland; NSRRC, Taiwan

**Keywords:** small-angle X-ray scattering, time-resolved SAXS, light activation

## Abstract

Beamline B21 at the Diamond Light Source synchrotron in the UK is a small-angle X-ray scattering (SAXS) beamline that specializes in high-throughput measurements *via* automated sample delivery systems. A system has been developed whereby a sample can be illuminated by a focused beam of light coincident with the X-ray beam. The system is compatible with the highly automated sample delivery system at the beamline and allows a beamline user to select a light source from a broad range of wavelengths across the UV and visible spectrum and to control the timing and duration of the light pulse with respect to the X-ray exposure of the SAXS measurement. The intensity of the light source has been characterized across the wavelength range enabling experiments where a quantitative measure of dose is important. Finally, the utility of the system is demonstrated *via* measurement of several light-responsive samples.

## Abstract 2

A system has been developed for irradiating samples with UV and visible light *in situ* during small-angle X-ray scattering data collection. The system is suitable for studying light-responsive samples such as photosurfactants, UV-hardening resins or light-active proteins.

## 1. Introduction

In the natural world, materials that change their structure in response to light result in sophisticated functionalities, including human vision and sunlight tracking in plants. Using this as inspiration, light-responsive materials are gathering interest in the scientific community for a wide range of applications spanning from soft robotics (Pilz da Cunha *et al.*, 2020 ▸), to energy storage (Zhang *et al.*, 2023 ▸), to drug delivery (Chander *et al.*, 2021 ▸). In comparison with other potential stimuli, light is particularly attractive as it can be applied externally and controlled for time, duration, wavelength, intensity and position of irradiation. Small-angle X-ray scattering (SAXS) is a powerful technique to study the nanoscale structure of materials and is widely employed to measure both soft condensed matter – including polymers, colloids and liquid crystals – as well as biological systems – such as proteins, peptides and DNA. However, when studying light-responsive materials, *ex situ* irradiation of the sample prior to loading and measurement can lead to inaccuracies as a result of sample relaxation from the light-activated state during the time taken for sample transfer and measurement. In addition to the time delay, the transfer process can also result in exposure to ambient light, which can act to further accelerate sample relaxation, as seen in azo­benzene photoswitches (Bandara & Burdette, 2012 ▸) and light-responsive proteins (Bannister *et al.*, 2019 ▸). This leads to ambiguity in the SAXS data as to whether the sample measured is still in the light-activated state.

To combat this, irradiation of the sample *in situ* can remove the time lag between irradiation and SAXS measurement, as well as preventing exposure to ambient light during sample transfer. Furthermore, *in situ* irradiation enables time-resolved data collection through stroboscopic or pump/probe irradiation and SAXS measurement (Burian *et al.*, 2020 ▸), unlocking access to structural information related to mechanisms of change that could not be obtained through simple before-and-after measurements. As such, numerous beamlines have developed an ability for time-resolved data collection to probe light-responsive systems, such as at the diffraction beamline ID09 at the European Synchrotron Radiation Facility (Wulff *et al.*, 2003 ▸), and the scattering beamline BioCARS at the Advanced Photon Source (Henning *et al.*, 2024 ▸). For light-responsive biological structures, this information about time-resolved conformational changes in solution could bridge the gap between fast, chemical changes observed using spectroscopic techniques and the static, before-and-after structures obtained using macromolecular crystallography (Bannister *et al.*, 2019 ▸; Berntsson *et al.*, 2019 ▸); whereas in soft-matter design, *in situ* light-irradiation has been used to deduct mechanistic information about formation and destruction of micelles (Lund *et al.*, 2016 ▸), variation in vesicle bilayer thickness (Ober *et al.*, 2022 ▸) or micelle morphology changes (Royes *et al.*, 2022 ▸). Beyond this, similar time-resolved experiments could help direct sample design for applications such as increasing energy storage, refining control over small-molecule encapsulation and release or maximizing geometrical changes in soft robotics.

In this work, we designed and implemented an easy-to-use, high-throughput system for *in situ* light-emitting diode (LED) irradiation during SAXS experiments at B21, Diamond Light Source (Cowieson *et al.*, 2020 ▸). An LED source is fibre-coupled to the SAXS sample environment, where it is reflected from a mirror to arrive co-incidentally at the sample position with the X-ray beam. Two different LED sources allow irradiation with 16 different wavelengths from 365 to 770 nm, but the flexible design means that the system is not limited to these sources. Furthermore, the simple fibre-optic design leads to an easy, ‘plug-and-play’ set-up, limiting experimental down-time and maintaining the high-throughput nature of B21. We demonstrate that this system can be used to study time-resolved changes in the self-assembly of light-responsive micelles, liquid crystals, lipids and polymers.

## 2. Experimental

### 2.1. Materials

The fibre-port collimator (effective focal length = 7.5 mm, anti-reflection range = 400–700 nm, waist diameter = 1.23 mm), uncoated plano-convex lens (diameter = 0.5 inches, focal length = 40 mm) and fused silica dielectric mirror (broadband coating = 400–750 nm, diameter = 7 mm, thickness = 2 mm) were purchased from Thorlabs. The coolLED pe-4000 was connected to a solarization-resistant fibre (core diameter = 455 µm, numerical aperture = 0.22, length = 2 m) provided by Ocean Insight. The high-power UV LED was supplied by Prizmatix (FC1-LED-365A) and connected to a polymeric fibre (core diameter = 1500 µm, numerical aperture = 0.5, length = 1 m, supplied by Prizmatix).

Kapton tape was supplied by RS Components. Polyimide capillaries were supplied by Spectrum Plastics Group. Quartz capillaries were supplied by Capillary Tube Supplies Ltd. Synthetic mica sheets were supplied by Great Wall Mineral (China). UV-penetrable tape was provided by Crystal Clear. 3-D printed sample sticks were made using *Formlabs Perform* printing software, on a Formlabs Form 2 printer in clear resin V2 or Resin V4.

The azo­benzene tri­methyl­ammonium bromide (AzoTAB) surfactant 4-hexyl­azo­benzene 4′-(oxybutyl) tri­methyl­ammonium bromide was synthesized by C. Blayo, as reported previously (Blayo *et al.*, 2018 ▸). Synthesized compounds and intermediates were characterized using ^1^H and ^13^C nuclear magnetic resonance (NMR) spectroscopy, high-resolution mass spectrometry (HRMS), Fourier transform infrared (FTIR) spectroscopy and melting-point analysis. AzoTAB was added to deionized water (50 m*M*) and shaken to form a micellar solution.

The neutral azo­benzene photosurfactant (AzoPS) 4-decyl-4′-(mono-tetra­ethyl­ene glycol) but­oxy azo­benzene (C_10_AzoC_4_E_4_) was synthesized by B. E. Jones, using a method from previous literature (Houston *et al.*, 2019 ▸). The compound was characterized using ^1^H and ^13^C NMR. To form a lyotropic liquid crystal (LLC), deionized water (30 wt%) was added to the AzoPS, the sample was heated to 60°C and mixed until homogeneous, before cooling to room temperature. The UV-cure polymer was Resin V4, supplied by Formlabs. The light-responsive anti-microbial lipid gel was composed of Pluronic F127 (35 wt%, supplied by Sigma Aldrich), surfactant-like peptide, R_4_F_4_ [1 wt%, custom-synthesized by C. J. C. Edwards-Gayle according to a method reported previously (Edwards-Gayle *et al.*, 2020 ▸)] and 2-hy­droxy-2-methyl­pio­phenone (1 wt%, supplied by Sigma Aldrich). Components were mixed together to give a homogeneous gel.

### 2.2. Beam characterization

The beam profile was taken using a Thorlabs camera beam profiler and analysed using the Thorlabs *Beam* 8.0 software. Power readings were taken using a Thorlabs PM400 optical power meter. The photodiode was supplied by Farnell and manufactured by Advanced Photonix (part number SLCD-61N8, 1.3 mm × 3.4 mm).

### 2.3. UV-vis absorption spectroscopy

UV-vis absorption measurements were recorded on a Perkin Elmer Lambda 650 UV spectrometer with a slit width of 2 nm and a scan speed of 266.75 nm min^−1^. Measurements were taken at 1 nm intervals from 800 to 200 nm. Quartz was measured against no background. All other samples were loaded onto a quartz slide and measured against a blank quartz slide reference.

### 2.4. Small-angle X-ray scattering

Small-angle X-ray scattering was carried out at beamline B21, Diamond Light Source. Samples were either loaded into quartz or polyimide capillaries manually or delivered automatically from a PCR strip into a quartz capillary using a liquid handling robot (Arinax/EMBL). The sample-to-detector distance was 3.7 m and the X-ray energy was 13.1 keV. The exposure temperature was set to 20°C for all samples, apart from the AzoTAB micelles, where the temperature was 25°C due to the low solubility of the sample at 20°C. During the experiments, the temperature of the sample exposure unit was monitored on the Arinax/EMBL software. Samples were first exposed to X-rays for a series of frames, and the number of frames that can be used before X-ray induced beam damage or clear sample change due to diffusion or reversal effects was determined using *ScÅtter* (https://www.bioisis.net/, R. P. Rambo). TTL signalling *via* a TFG2 time frame generator (Daresbury Laboratories) was used to control light irradiation periods, followed by SAXS frames; there was a minimum delay of 100 ms between light irradiation and SAXS acquisition to allow for signalling. The background was manually subtracted and frames were integrated using *ScÅtter*.

The AzoTAB micelle sample (10 µL) was automatically loaded using the liquid-handling robot into a quartz capillary (1.5 mm diameter) which is fixed inside the sample exposure unit. Samples were held in ‘static mode’ during UV irradiation and SAXS measurement. The sample was irradiated for 80 min with UV light (coolLED, 365 nm, 3.47 W m^−2^). Three SAXS frames of 500 ms each were taken at 10 min intervals and integrated to give the timestamps displayed.

The AzoPS LLC, UV-cure polymer and light-responsive anti-microbial lipid gel were all injected into disposable quartz capillaries (1.5 mm in diameter). The capillaries were held in a 3-D printed lollystick and manually loaded into the beamline for SAXS measurements. For the AzoPS LLC sample, the capillary was irradiated with UV (Prizmatix, 365 nm, 3.40 W m^−2^) light for 4 h, with three SAXS frames of 500 ms taken every 10 min. Subsequently, the sample was left to relax in the dark and three SAXS frames of 500 ms were taken every 30 min for 5 h. For the UV-cure polymer, the samples were irradiated for 0 s, 10 s, 60 s or 20 min with UV light (Prizmatix, 365 nm, 120 W m^−2^), before measuring five X-ray frames of 250 ms each. A reference sample was irradiated in a UV light box outside the beamline (365 nm, 60 min, 8 W m^−2^), and measured over 20 frames of 250 ms each. For the light-responsive antimicrobial lipid gel, samples were irradiated with 0, 5, 10, 15 and 30 min of green (coolLED, 550 nm, 33 W m^−2^) light, before measuring 20 X-ray frames of 250 ms each.

### 2.5. SAXS fitting

For SAXS analysis, *OriginPro* 2021b (OriginLab, USA) was used to fit linear regions or to pick the peak positions. Calculations of the real-space distances from structure factor peaks were calculated using the equation *d* = 2π/*q*, where *d* is the real-space distance and *q* is the *q* position of the centre of the peak maximum.

For AzoTAB micelles, the data were fitted using *SASFit* (version 0.94.11) (Breßler *et al.*, 2015 ▸). The first 50 data points were removed and a linear, horizontal background was set to an appropriate value. The data after 0 min of UV irradiation were fitted to an ellipsoidal core-shell cylindrical micelle structure, with a Gaussian distribution around the polar radius to incorporate polydispersity into the model. Data were fitted to the polar radius, ellipsoidal radius, length, shell thickness and scattering length density. The data after 80 min of UV irradiation were fitted to an ellipsoidal core-shell structure, with a Gaussian distribution around the polar radius to incorporate polydispersity into the model. Data were fitted to the polar radius, ellipsoidal radius, shell thickness and scattering length density. A structure factor was also required to model the charged micelle interactions. To achieve this, a Hayter-Penfold Rescaled Mean Spherical Approximation (RMSA) MSA model was fitted to the variables: hard-sphere radius, charge and volume fraction of micelles.

## 3. Development of the *in situ* LED irradiation system

### 3.1. Layout design

B21 is a high-throughput SAXS beamline, meaning that a key design-focus was to create a ‘plug-and-play’ irradiation system, with minimum instrument down-time and intervention in the sample environment before measurement. The B21 sample environment consists of a temperature-controlled sample exposure unit (SEU), provided by Arinax/EMBL (Round *et al.*, 2015 ▸). This supports the loading of samples *via* an automated liquid-handling robot, high-performance liquid chromatography (HPLC) or manually, using a multi-purpose sample cell for high-viscosity liquids, gels or solid samples (Edwards-Gayle *et al.*, 2021 ▸). The sample capillary and flow-device inputs meant that light irradiation along the axis of the sample was not feasible. A sample-viewing camera positioned perpendicular to the X-ray beam meant that top-down irradiation was also not possible. This left irradiation parallel to the X-ray beam as the only available option.

**Figure 1:**
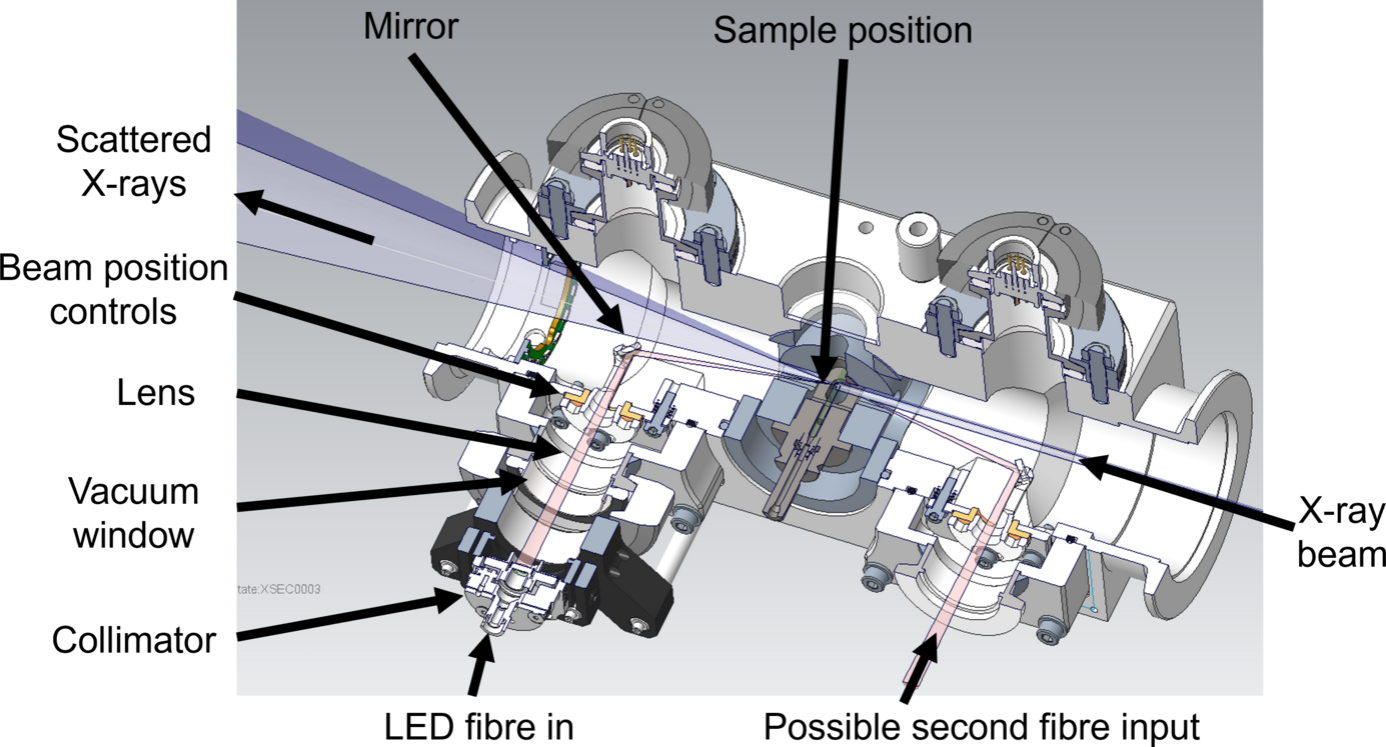
Design for the *in situ* irradiation environment. The X-ray beam is incident from the right. The LED fibre input is downstream from the sample position; the light beam travels through a series of focusing optics before being reflected, parallel to the X-ray beam, onto the sample. The design has flexibility for a second fibre input upstream from the sample position, but this is not currently in use.

In the final design, an LED light is fed into the SEU through a fibre-optic input, which is exterior to the vacuum of the sample environment (Fig. 1 ▸). It is passed through a collimator, vacuum window, focusing lens and then reflected off a mirror onto the sample, parallel to the X-ray beam. The beam position in relation to the sample can be imaged using an on-axis camera positioned on the opposite side of the sample to the LED input. To aid imaging using this camera, a white, LED illumination ring was added to the inside of the sample chamber. This allows backlighting of the sample for inspection, as well as to detect the position of the light beam. Since the X-ray beam is centred before the experiment to the centre of the sample capillary, it can be assumed that a central position of the LED beam on the capillary will result in good overlap between the light and X-ray beams. The design has flexibility to include a second irradiation route from upstream of the sample position (Fig. 1 ▸) which could allow two-side illumination; however, this is not currently in use.

Two different light sources are available to input into the system: a pE-4000 (coolLED) multi-wavelength device, with 16 selectable wavelengths (365–770 nm) and up to four available simultaneously, and a high-power UV source at 365 nm (Prizmatix). The wide range of wavelengths provided by the coolLED means that the system is versatile to a number of different experiments, with different requirements, without the need to re-configure the set-up between them and therefore maintains the high-throughput nature of B21. High-power UV light was chosen to specifically study high-concentration materials containing an azo­benzene-type group, which is one of the most widely used photoswitches. However, the fibre-port input means that the design is not limited to these sources, and it can be integrated with many different LED or laser sources, depending on the requirements for the experiment.

### 3.2. Beam position, shape and size

B21 is a bending-magnet beamline with a beam size of 1102 µm × 240 µm at the sample position (Cowieson *et al.*, 2020 ▸). Whilst a higher LED focus and smaller spot size would increase the irradiance (power transferred per unit area) delivered to the sample, a spot size of <1 mm is undesirable as this is less than the width of the X-ray beam at the sample position. This would mean that not all the sample being measured by SAXS is being irradiated. Furthermore, the nature of SAXS samples, which are often liquids or gels, means that sample diffusion into and out of both the X-ray and LED beams is significant on the timescales of sample measurement (∼20 s). This means that it is desirable to have an LED beam that is larger than the X-ray beam at the sample position, preventing diffusion of un-irradiated sample into the X-ray path which would ‘blur’ the image of the irradiated state.

The LED optics section was designed to allow adjustment of the LED beam focus and position. The reflecting mirror is attached to an optic holder that allows adjustment in *x* translation, yaw (around *y*) and roll (around *z*) using a combination of three grub screws (Fig. S1 of the supporting information). Pitch (around *x*) is also adjustable through rotation of the whole optic holder (Fig. S1). The total path length from the fibre-port input to the sample position is 143.19 mm. The fibre-optic input is collimated using a fibre-port achromatic collimator with a SubMiniature, version A (SMA), connection which enables easy set-up and versatility in the input light source. *x*, *y* and *z* controls on the fibre port allow external control over the position of the light beam as it is focused onto the mirror. The collimator and controls lie outside of the vacuum window, which allows fine adjustments of the light beam to be made easily, without having to vent the sample environment and dismantle the optics section to gain access to the optic holder where the mirror sits. The light beam is focused using an uncoated plano-convex lens.

**Figure 2:**
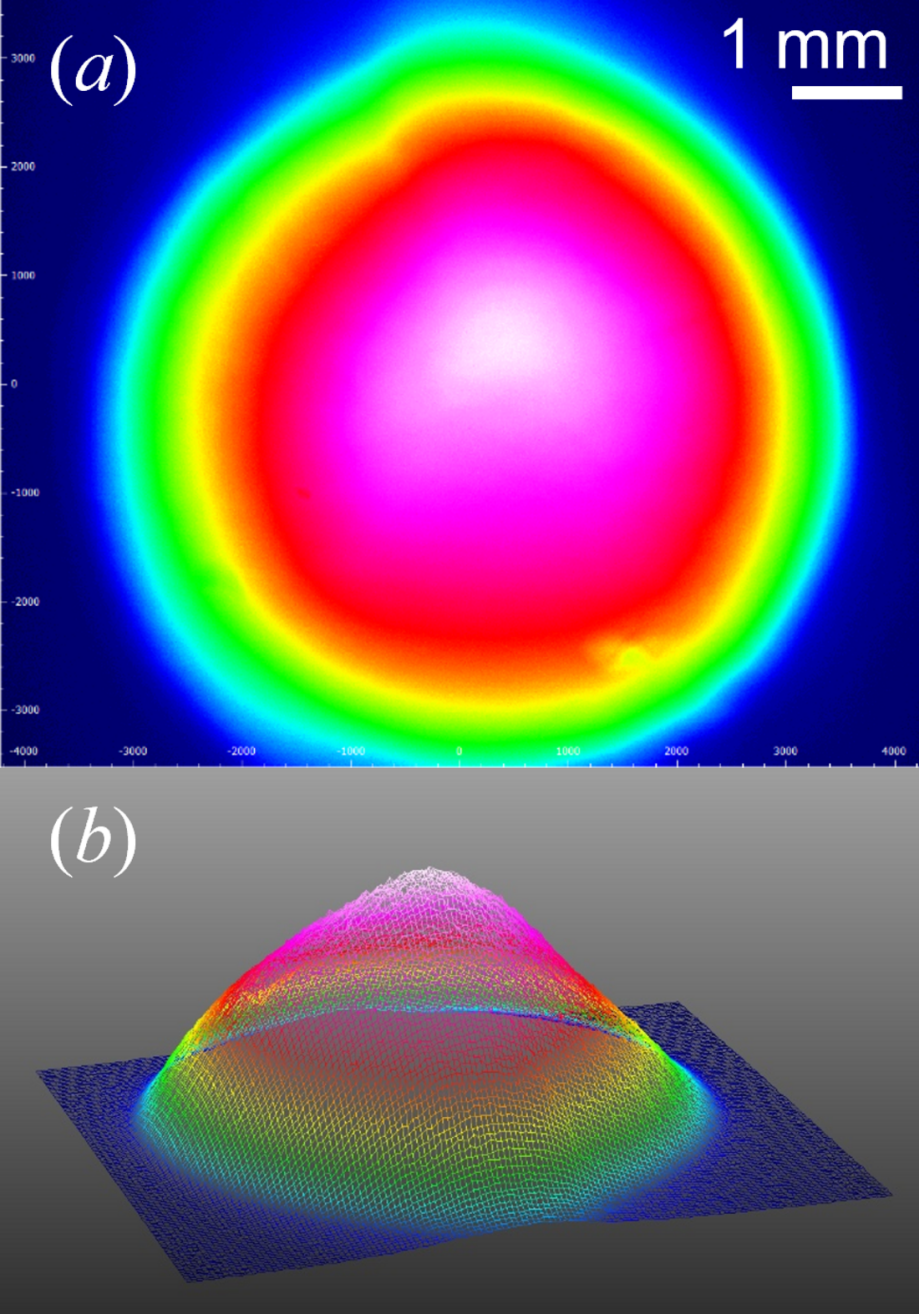
(*a*) 2-D and (*b*) 3-D beam profile images showing a spherical shape to the light beam at the sample position. The light source is the coolLED light at a wavelength of 365 nm and 35% of the maximum power rating.

The shape of the LED beam was determined using a beam profiler at the sample position on a 3-D printed model of the SEU. The images show that the optics section and mirror do not cause significant distortion of the beam shape, producing an elliptical spot (Fig. 2 ▸). The full width at half-maximum for the light beam in the *x* and *y* directions were determined from a Gaussian fit to the *x* and *y* profiles using the Thorlabs *Beam* 8.0 software (Fig. S2 of the supporting information). These are 4.5 mm (*x*) and 3.6 mm (*y*), giving a total beam area of 50.9 mm^2^. The sample capillary is 1.5 mm in diameter, meaning that the beam fully encompasses the sample in this direction. Having a greater width in the *x* direction allows for a greater region to be irradiated where the X-ray beam is larger and where the capillary is longer.

### 3.3. Characterizing LED irradiance

**Figure 3:**
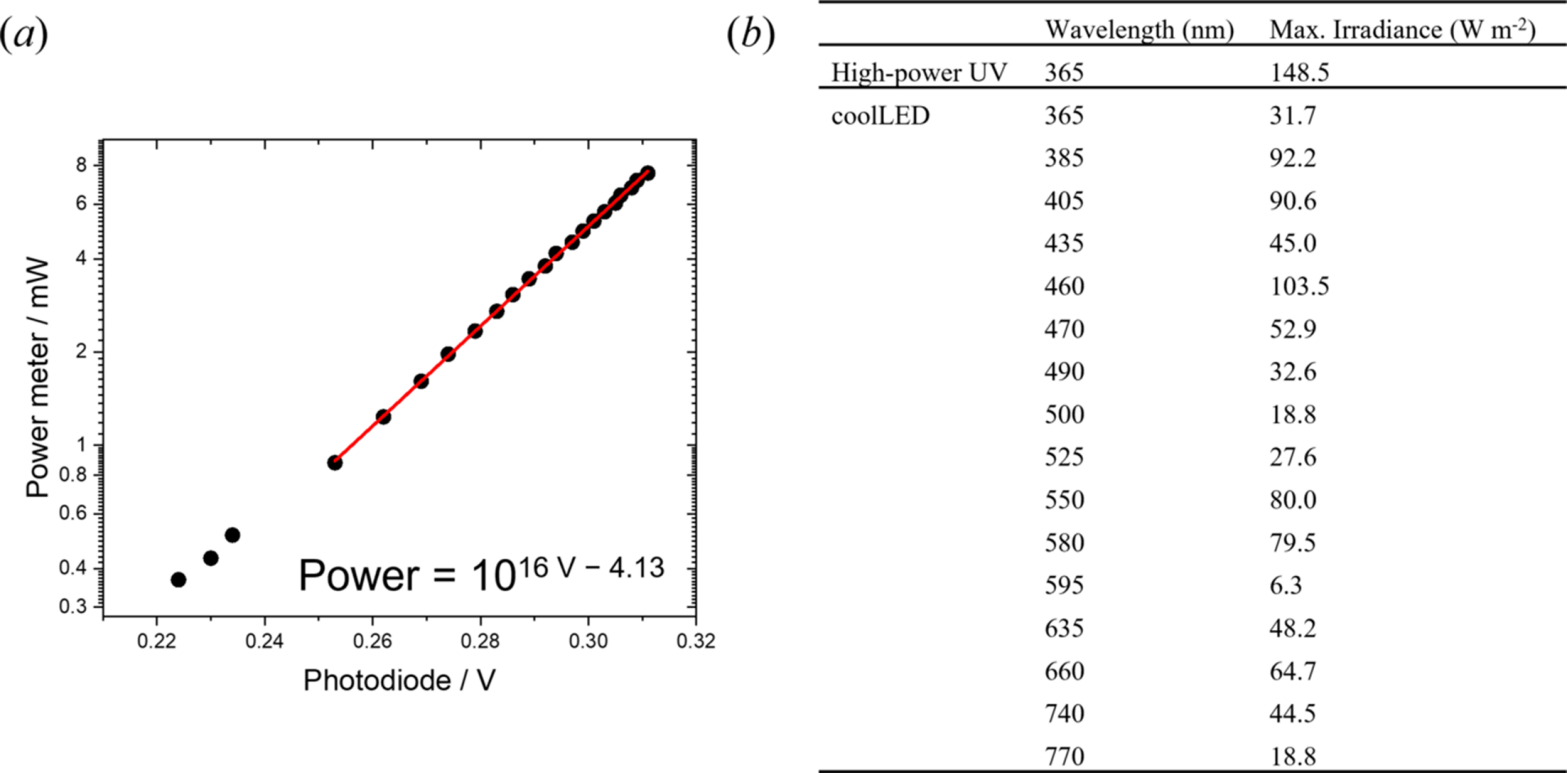
(*a*) Calibration curve used to determine the irradiance from the high-power UV source on the sample by comparing the voltage (V) from a photodiode on the sample stick to the equivalent power reading (mW) from a photothermal power meter. (*b*) Maximum irradiance at the sample position as a function of wavelength for the coolLED and high-power UV (Prizmatix) light sources.

The power output of the LED sources was next optimized. For the multi-wavelength coolLED system, the power output was maximized by increasing the core diameter of the connecting quartz fibre optic cable; the largest diameter tested was 455 µm, with a numerical aperture (NA) of 0.22 (Fig. S3 of the supporting information). For the high-power UV source, a polymeric fibre (core diameter = 1500 µm, NA = 0.5) achieved a higher power output (96 mW *cf.* 5.65 mW) than the highest core diameter quartz fibre. The resulting power delivered to the sample by this high-power LED is 4.7 times larger than that provided by the coolLED system at 365 nm [Fig. 3 ▸(*b*)]. It is worth noting that there is a variation in the power delivered from the coolLED system depending on the wavelength selected. This variation is not systematic with increasing wavelength [Fig. 3 ▸(*b*)], suggesting this is due to differing efficiencies of LEDs within the device, rather than an effect of the optics system.

A method to rapidly determine the irradiance at the sample position before an experiment was developed. The limited space in the sample environment means that measurement of the LED power using a photothermal power meter would require lengthy dismantling of the temperature core, resulting in a large down-time before experiments. Instead, a small photodiode was loaded onto a 3-D printed sample stick (Edwards-Gayle *et al.*, 2021 ▸) using Kapton tape, that can be easily inserted where the sample will sit. The output voltage from the photodiode can be used to calculate the irradiance on the sample, using equations determined from calibration curves with photothermal power meter readings. These readings were obtained after dismantling the temperature core and inserting the photothermal power meter at the sample position. The calibration curves were attained by sequentially increasing the power rating on the light source and noting the output readings from the photothermal power meter at the sample position, and the corresponding voltage from the photodiode loaded onto a sample holder. Plots of the logarithm of the power meter reading versus the photodiode reading give a straight-line dependence in the high-power limit [Fig. 3 ▸(*a*)]. This was used to derive equations to calculate the power incident on the sample using the photodiode reading [Table S1 and equations (S1) and (S2) of the supporting information]. The wavelength dependence on the output from the LEDs and the photodiode means that separate calibration curves and conversion equations are required for each wavelength (Figs. S4 and S5 of the supporting information). The power incident on the sample can then be converted to an irradiance by dividing by the approximate beam size (50.9 mm^2^). This can be directly compared with laboratory sources used for previous experiments, enabling the time for photo-activation or photo-conversion using this system to be predicted to inform the time between SAXS measurements and irradiation profiles in subsequent experiments.

### 3.4. Sample environment design

**Figure 4:**
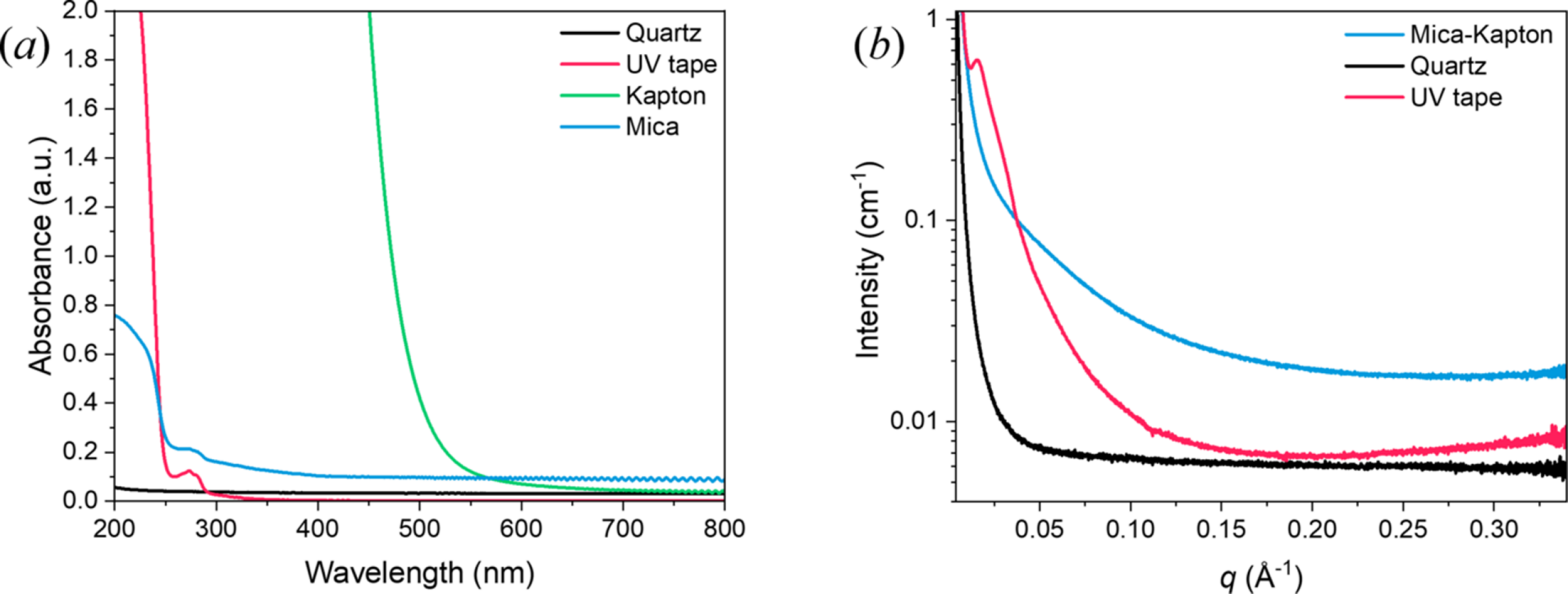
(*a*) UV-vis absorption spectra for the sample environment materials. Low absorption for quartz, UV tape, and mica above 300 nm, deem them suitable materials for light-activated experiments. (*b*) SAXS curves for the sample environment materials.

SAXS measurements of light-activated samples require sample environments that are light-penetrable, as well as having a low SAXS background scatter. At B21, low-viscosity liquids are loaded by injecting them into a quartz capillary using an automated liquid-handling robot or from an HPLC column. Quartz has a low absorbance across the UV-vis wavelength range [Fig. 4 ▸(*a*)], meaning this loading environment is suitable for light-activated experiments. Previously, high-viscosity or solid samples have been manually loaded onto a custom, 3-D printed multi-purpose sample (MPS) stick using a Kapton-tape seal (Edwards-Gayle *et al.*, 2021 ▸). However, Kapton has a high absorbance at wavelengths <500 nm [Fig. 4 ▸(*a*)], meaning it is unsuitable to seal samples for light-activated experiments. To combat this, three different solutions for MPS loading were developed: a combination of a synthetic mica window (facing the light source) and Kapton encapsulating tape (behind the sample); UV-penetrable tape; and manual injection into a quartz capillary. The combination of mica and Kapton was chosen to use the high light-transmission of mica on the side facing the light source, and using Kapton tape behind the sample, due to its lower cost. These three options allow flexibility depending on the specific viscosity of the sample to be loaded. All these materials have a low absorbance above 300 nm [Fig. 4 ▸(*a*)], meaning they are suitable for this irradiation system, where the minimum wavelength is 365 nm.

As well as light-transmissibility, the sample environment must also exhibit low scattering in the small-angle range to minimize the background for data collection. Of the materials tested, the quartz capillary displays the lowest background scatter [Fig. 4 ▸(*b*)]; however, for high-viscosity samples the mica–Kapton or UV tape sample environments may still be preferable due to ease of loading and lack of high-shear effects which may be caused by capillary filling. It is worth noting that there is a small peak in the UV tape SAXS curve at *q* = 0.015 Å^−1^, meaning consideration of the range of interest from the sample scattering is needed before deciding on the best sample environment to choose.

An additional challenge to the sample environment design for light-activated experiments is sample dehydration. The nature of these experiments tracks the sample over time as it is exposed to light. This results in experiments that can be much longer than standard SAXS measurements (up to minutes or hours *cf.* ∼20 s for a single sample), meaning that dehydration of liquid or gel samples is a concern. It was found that the mica–Kapton design was particularly susceptible to dehydration due to capillary forces that pull the sample from the hole and into small gaps between the mica sheet and sample stick, or between cracks in the mica, during the experiment [Fig. S6(*a*) of the supporting information]. In this respect, the UV-penetrable tape worked better, due to a better seal with the sample stick [Fig. S6(*b*) of the supporting information]. It is thought that over long experiments additional dehydration of the sample can result from water being drawn into the 3-D printed sample stick itself, due to inherent porosity in the polymer structure. This was visible as a tainting of the polymer sample stick with the orange colour of the sample over the course of the 4 h experiment [Fig. S6(*b*) of the supporting information]. To combat this, the 3-D printed sample stick can be replaced with an aluminium sheet containing a hole for the sample, which is less likely to intake water.

## 4. Data collection using the *in situ* irradiation system

**Figure 5:**
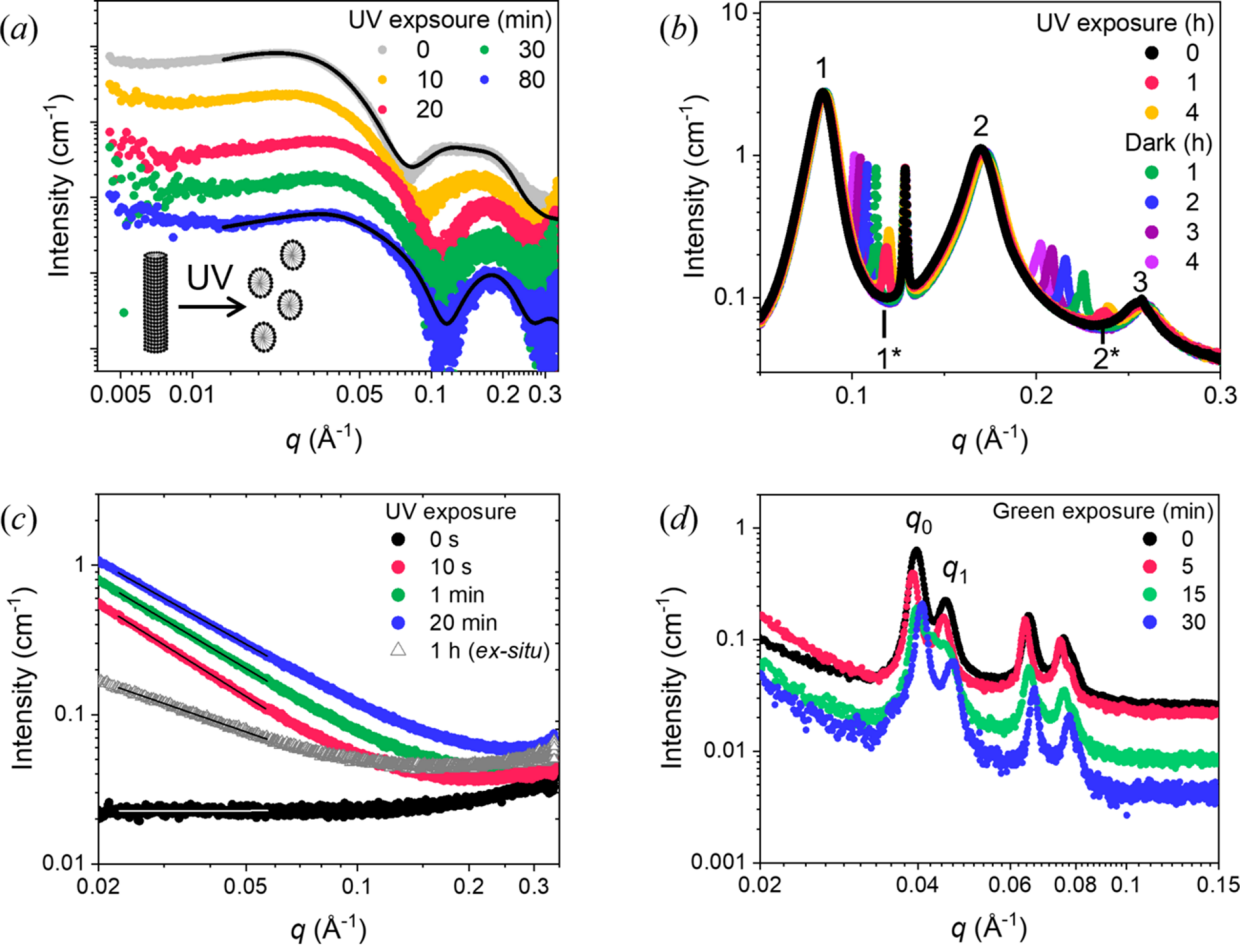
SAXS data collected using the *in situ* LED irradiation system showing: (*a*) changes to micelle shape on irradiation of AzoTAB surfactants with UV light; (*b*) changes to the lyotropic liquid crystal structure formed from AzoPS surfactants on irradiation with UV light and relaxation in the dark; (*c*) dynamics of a UV-induced polymerization reaction; and (*d*) structural changes in an anti-microbial lipid gel on irradiation with green light. Fits to the data are depicted as solid black or white lines. Note that the data in (*a*) have been shifted vertically for clarity.

Data collection is controlled using time-to-live (TTL) pulses that can be used to trigger the X-ray fast shutter and detector, as well as the LED system. This allows integration of irradiation with SAXS measurements, enabling a versatile range of possible sequences of irradiation, as well as cycling between different wavelengths. The system was tested with four different example samples. Firstly, the high-power UV source was used to show a change in the scattering from micelles of light-responsive azo­benzene tri­methyl­ammonium bromide (AzoTAB) surfactants at 50 m*M* in water [Fig. 5 ▸(*a*)]. Azo­benzene in the surfactant molecules isomerizes under UV light, from a *trans* to a *cis* isomer, which leads to a change in the shape and polarity of the AzoTAB. This has been shown previously, using *ex situ* irradiation, to have a knock-on effect for the micelle shape (Blayo *et al.*, 2018 ▸). Here, *in situ* UV irradiation shows time-resolved changes in the scattering, which can be attributed to structural changes within the AzoTAB micelles due to isomerization of the azo­benzene group [Fig. 5 ▸(*a*)]. Changes to the structure are visible within 10 min of UV irradiation and reach an equilibrium where no further changes are observed after around 20 min of irradiation, due to the formation of a photostationary state where there is no change in the percentage *cis* isomers present in the sample. Fits to the SAXS data show that this can be described by a change in micelle shape from ellipsoidal cylinders to prolate ellipsoids (Table S2 of the supporting information). This favouring of a morphology with a higher curvature can be expected due to the increase in tail group volume of the AzoTAB on isomerization (Blayo *et al.*, 2018 ▸), increasing the surfactant critical packing parameter and favouring the break-up of the cylindrical micelles in favour of smaller, higher-curvature ellipsoids.

The high-power UV source was next used to investigate a lyotropic liquid crystal (LLC) formed from a neutral azo­benzene photosurfactant (AzoPS) at a concentration of 70 wt% in water on irradiation with UV light to induce isomerization. The AzoPS forms sharp, Bragg diffraction peaks in the native, *trans* isomer indicating the formation of a highly ordered, LLC structure [Fig. 5 ▸(*b*)]. The *q* positions of the peak centres are in a ratio of 1:2:3, characteristic of a lamellar LLC phase, with an interlamellar (*d*) spacing of 75 Å, as calculated using the position of the primary Bragg peak. On irradiation with UV light, a second lamellar phase forms, as shown by the formation of two new peaks [1* and 2*, Fig. 5 ▸(*b*)] at higher *q* values than those in the original, *trans* isomer. The *d* spacing in the new phase is 53 Å, showing that the isomerized, *cis* isomer packs more tightly in the lamellar phase. This is expected due to the shorter end-to-end length of the surfactant on isomerization, caused by the bending in the tail, which has resulted in similar LLC contraction in previous work (Jones *et al.*, 2022 ▸). On subsequent storage of the irradiated LLC in the dark, the peaks shift back to lower *q* values, indicating a swelling of the lamellar phase. This is to be expected due to gradual thermal relaxation of the AzoPS to the original, *trans* isomer, which will increase the surfactant end-to-end length and increase the inter-lamellar spacing. *In situ* irradiation therefore enables mechanistic insight into structural changes in these systems, which could expand the understanding over previous experiments where such illumination was not possible (Houston *et al.*, 2019 ▸; Jones *et al.*, 2024 ▸). Furthermore, azo­benzene photoswitches have been shown to reverse isomerize under X-ray irradiation (Ober *et al.*, 2022 ▸), meaning *in situ* irradiation is also beneficial to ensure the photostationary state is being probed through continuous irradiation until no further structural changes are observed.

Beyond light-responsive surfactants, the system was also used to track changes in the scattering during UV-induced polymerization [Fig. 5 ▸(*c*)]. On *in situ* UV irradiation, a change in the power-law dependence (*q*^−*n*^) of the scattering curve at low *q* (0.017–0.05 Å^−1^) is seen. The curves were fitted to straight lines in this region, showing a transition from a slope of 0 at 0 min irradiation to −1.6 at 10 s, −1.5 at 1 min and −1.4 at 20 min. In comparison with this, a sample that had been irradiated for 60 min *ex situ* displayed a power-law of −0.9. This suggests that the sample is transitioning from monomer units that are too small to scatter significantly at time zero to polymeric, mass fractal structures which appear from 10 s of UV irradiation. The decrease in power-law exponent over time could indicate a mechanism of swelling of the polymer chains on increasing irradiation time as they become closer to their final polymer form (Beaucage, 1996 ▸).

The final example is a lipid gel that contains anti-microbial peptides that displays structural changes on irradiation with green light [Fig. 5 ▸(*d*)]. Light irradiation led to a shift in the structure-factor peaks to higher *q* values, corresponding to a change in real-space distance from 158 to 154 Å for the primary peak (*q*_0_) and from 140 to 134 Å for the secondary peak (*q*_1_), indicating a shrinking of the gel matrix. Furthermore, a change in the background slope, from −1.7 to −2.0, could indicate a slight collapse of the underlying mass fractal structure in the lipid gel (Beaucage, 1996 ▸). All examples show significant benefits in terms of dynamic structural information than could be achieved using *ex situ* irradiation and subsequent SAXS measurement.

## 5. Summary and outlook

In summary, we have developed an optics system for the sample environment at B21, Diamond Light Source, that can integrate UV-, vis- or infrared-irradiation experiments with SAXS. The ‘plug-and-play’ fibre-optic input requires little time for experimental set-up and alignment, maintaining the high-throughput nature of B21. Furthermore, the design can easily be integrated with LED or laser sources of other wavelengths through an SMA connection or manual alignment through the vacuum window onto the mirror. This expands the potential at B21 to a variety of light-activated or temperature-jump experiments to understand the time-resolved dynamics of structural changes in surfactants, gels, colloids, polymers or proteins.

## Supplementary Material

Sections S1 to S5 including Tables S1 and S2 and Figures S1 to S6. DOI: 10.1107/S1600577524003205/ju5061sup1.pdf

## References

[bb1] Bandara, H. M. D. & Burdette, S. C. (2012). *Chem. Soc. Rev.* **41**, 1809–1825. 10.1039/c1cs15179g 22008710

[bb2] Bannister, S., Böhm, E., Zinn, T., Hellweg, T. & Kottke, T. (2019). *Struct. Dyn.* **6**, 034701. 10.1063/1.5095063 PMC6588521 31263739

[bb3] Beaucage, G. (1996). *J. Appl. Cryst.* **29**, 134–146.

[bb4] Berntsson, O., Rodriguez, R., Henry, L., Panman, M. R., Hughes, A. J., Einholz, C., Weber, S., Ihalainen, J. A., Henning, R., Kosheleva, I., Schleicher, E. & Westenhoff, S. (2019). *Sci. Adv.* **5**, eaaw1531. 10.1126/sciadv.aaw1531 PMC6636987 31328161

[bb5] Blayo, C., Houston, J. E., King, S. M. & Evans, R. C. (2018). *Langmuir*, **34**, 10123–10134. 10.1021/acs.langmuir.8b02109 30071720

[bb6] Breßler, I., Kohlbrecher, J. & Thünemann, A. F. (2015). *J. Appl. Cryst.* **48**, 1587–1598. 10.1107/S1600576715016544 PMC4603274 26500467

[bb7] Burian, M., Marmiroli, B., Radeticchio, A., Morello, C., Naumenko, D., Biasiol, G. & Amenitsch, H. (2020). *J. Synchrotron Rad.* **27**, 51–59. 10.1107/S1600577519015728 PMC6927520 31868736

[bb8] Chander, N., Morstein, J., Bolten, J. S., Shemet, A., Cullis, P. R., Trauner, D. & Witzigmann, D. (2021). *Small*, **17**, 2008198. 10.1002/smll.202008198 33880882

[bb9] Cowieson, N. P., Edwards-Gayle, C. J. C., Inoue, K., Khunti, N. S., Doutch, J., Williams, E., Daniels, S., Preece, G., Krumpa, N. A., Sutter, J. P., Tully, M. D., Terrill, N. J. & Rambo, R. P. (2020). *J. Synchrotron Rad.* **27**, 1438–1446. 10.1107/S1600577520009960 PMC7467336 32876621

[bb10] Edwards-Gayle, C. J. C., Barrett, G., Roy, S., Castelletto, V., Seitsonen, J., Ruokolainen, J. & Hamley, I. W. (2020). *ACS Appl. Bio Mater.* **3**, 1165–1175. 10.1021/acsabm.9b00894 PMC7147261 32296775

[bb11] Edwards-Gayle, C. J. C., Khunti, N., Hamley, I. W., Inoue, K., Cowieson, N. & Rambo, R. (2021). *J. Synchrotron Rad.* **28**, 318–321. 10.1107/S1600577520013831 PMC7842227 33399583

[bb12] Henning, R. W., Kosheleva, I., Šrajer, V., Kim, I.-S., Zoellner, E. & Ranganathan, R. (2024). *Struct. Dyn.* **11**, 014301. 10.1063/4.0000238 PMC10834067 38304444

[bb13] Houston, J. E., Kelly, E. A., Kruteva, M., Chrissopoulou, K., Cowieson, N. & Evans, R. C. (2019). *J. Mater. Chem. C.* **7**, 10945–10952.

[bb551] Jones, B. E., Greenfield, J. L., Cowieson, N., Fuchter, M. J. & Evans, R. C. (2024). *J. Am. Chem. Soc.* **146**, 12315–12319. 10.1021/jacs.4c02709 PMC11082889 38683357

[bb14] Jones, B. E., Kelly, E. A., Cowieson, N., Divitini, G. & Evans, R. C. (2022). *J. Am. Chem. Soc.* **144**, 19532–19541. 10.1021/jacs.2c08583 PMC9619397 36222426

[bb15] Lund, R., Brun, G., Chevallier, E., Narayanan, T. & Tribet, C. (2016). *Langmuir*, **32**, 2539–2548. 10.1021/acs.langmuir.5b04711 26928121

[bb16] Ober, M. F., Müller-Deku, A., Baptist, A., Ajanović, B., Amenitsch, H., Thorn-Seshold, O. & Nickel, B. (2022). *Nanophotonics*, **11**, 2361–2368. 10.1515/nanoph-2022-0053 PMC11636378 39678084

[bb17] Pilz da Cunha, M., Debije, M. G. & Schenning, A. P. H. J. (2020). *Chem. Soc. Rev.* **49**, 6568–6578. 10.1039/d0cs00363h 32779649

[bb18] Round, A., Felisaz, F., Fodinger, L., Gobbo, A., Huet, J., Villard, C., Blanchet, C. E., Pernot, P., McSweeney, S., Roessle, M., Svergun, D. I. & Cipriani, F. (2015). *Acta Cryst.* D**71**, 67–75. 10.1107/S1399004714026959 PMC4304687 25615861

[bb19] Royes, J., Bjørnestad, V. A., Brun, G., Narayanan, T., Lund, R. & Tribet, C. (2022). *J. Colloid Interface Sci.* **610**, 830–841. 10.1016/j.jcis.2021.11.133 34887060

[bb20] Wulff, M., Plech, A., Eybert, L., Randler, R., Schotte, F. & Anfinrud, P. (2003). *Faraday Disc.* **122**, 13–26. 10.1039/b202740m 12555847

[bb21] Zhang, L., Liu, H., Du, Q., Zhang, G., Zhu, S., Wu, Z. & Luo, X. (2023). *Small*, **19**, 2206623. 10.1002/smll.202206623 36534833

