# Supplementary material for: Developing an in situ LED irradiation system for small-angle X-ray scattering at B21, Diamond Light Source
Source: J Synchrotron Radiat. 2024 May 31;31(Pt 4):763–70. doi: 10.1107/S1600577524003205 (PMC11226168; doi:10.1107/S1600577524003205)
Supplement: Supplementary file 1 [file s-31-00763-sup1.pdf]

JOURNAL OF SYNCHROTRON RADIATION

Volume 31 (2024)

Supporting information for article:

Developing an *in-situ* LED irradiation system for small-angle X-ray scattering at B21, Diamond Light Source

Beatrice E. Jones, Ann Fitzpatrick, Kieran Fowell, Charlotte J. C. Edward-Gayle, Nikul Khunti, Katsuaki Inoue, Steven Daniels, Eugene Williams, Camille Blayo, Rachel C. Evans and Nathan Cowieson

S1. Beam position controls

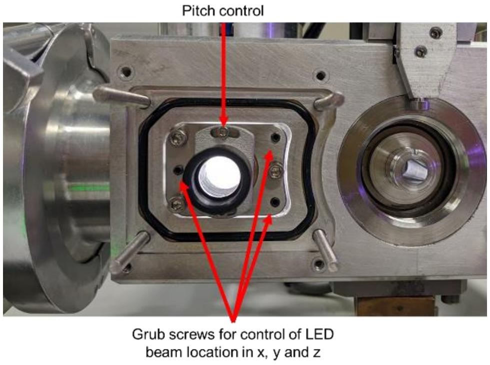

Figure S1 Positions of grub screws and pitch control of the optic holder, used to move the mirror and position of the LED beam in x, y and z directions.

S2. Beam profile

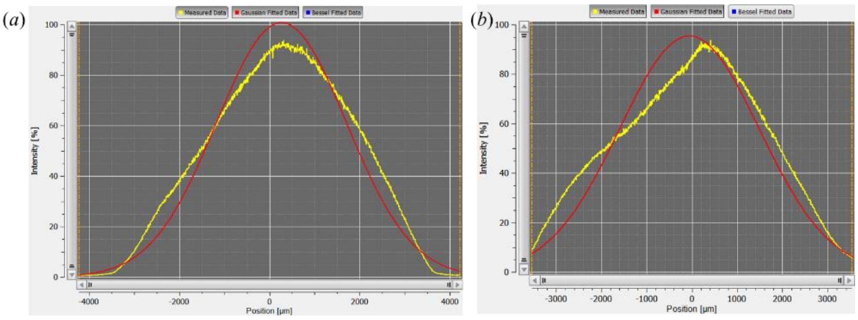

Figure S2 **(a)** x and **(b)** y profiles for the light beam at the sample position (yellow). Plots were fitted to Gaussian curves (red) using the Thorlabs Beam 8.0 software.

S3. Power measurements and calibration

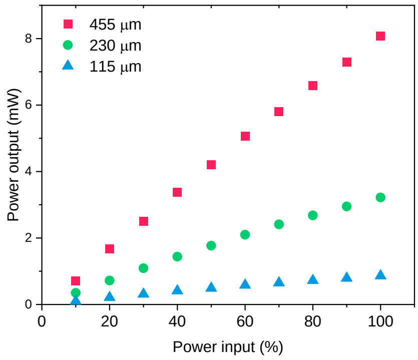

Figure S3 Power output (measured using a photothermal power meter) vs. the input intensity (%) for the coolLED at 365 nm on increasing the fibre core diameter. The fibre was placed at a distance of 1 cm from the power meter, without the focussing optics.

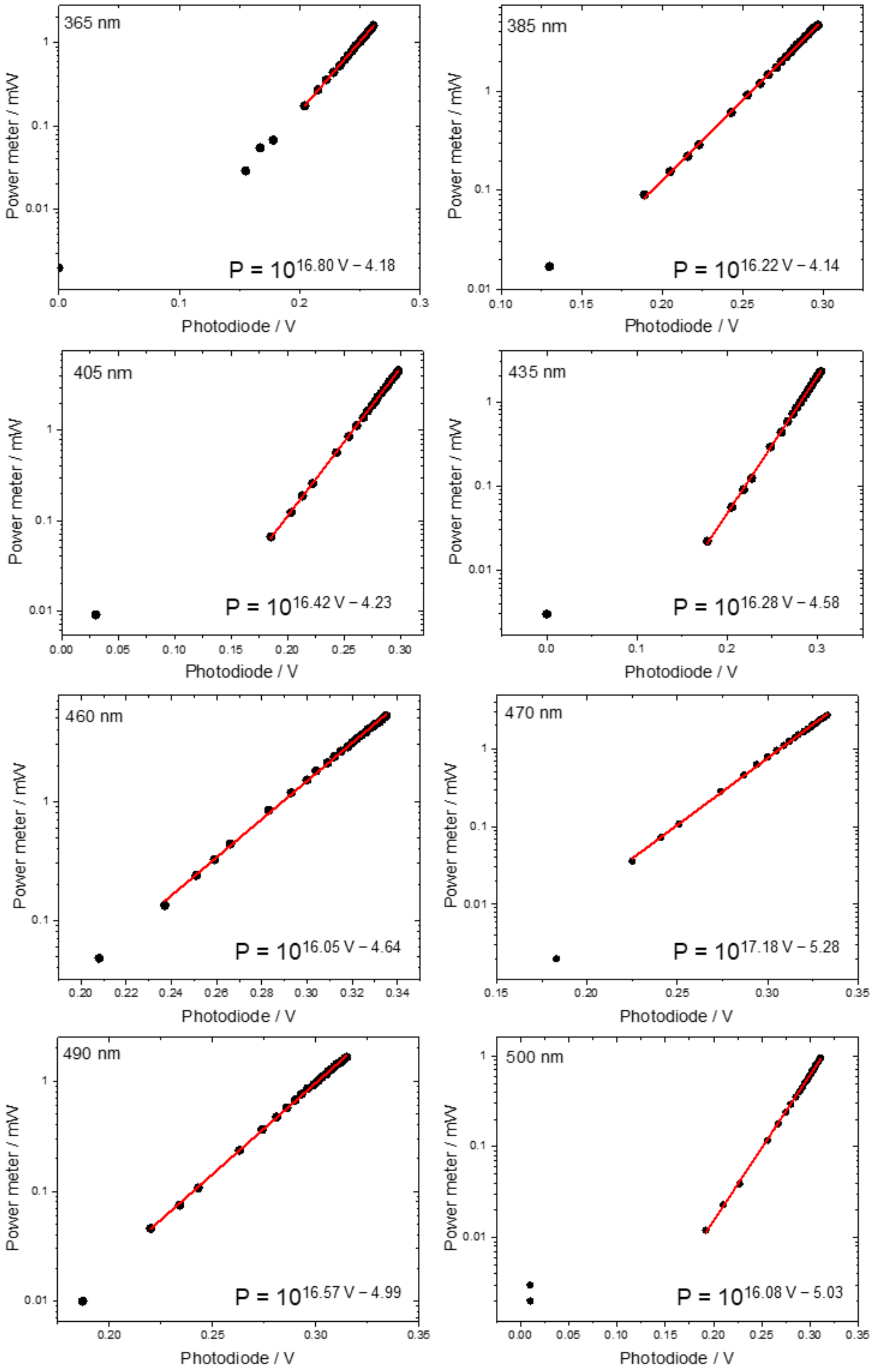

Figure S4 Calibration curves used to determine the conversion equations between the *in-situ* photodiode readings and the absolute power at different wavelengths (365-500 nm) for the coolLED system.

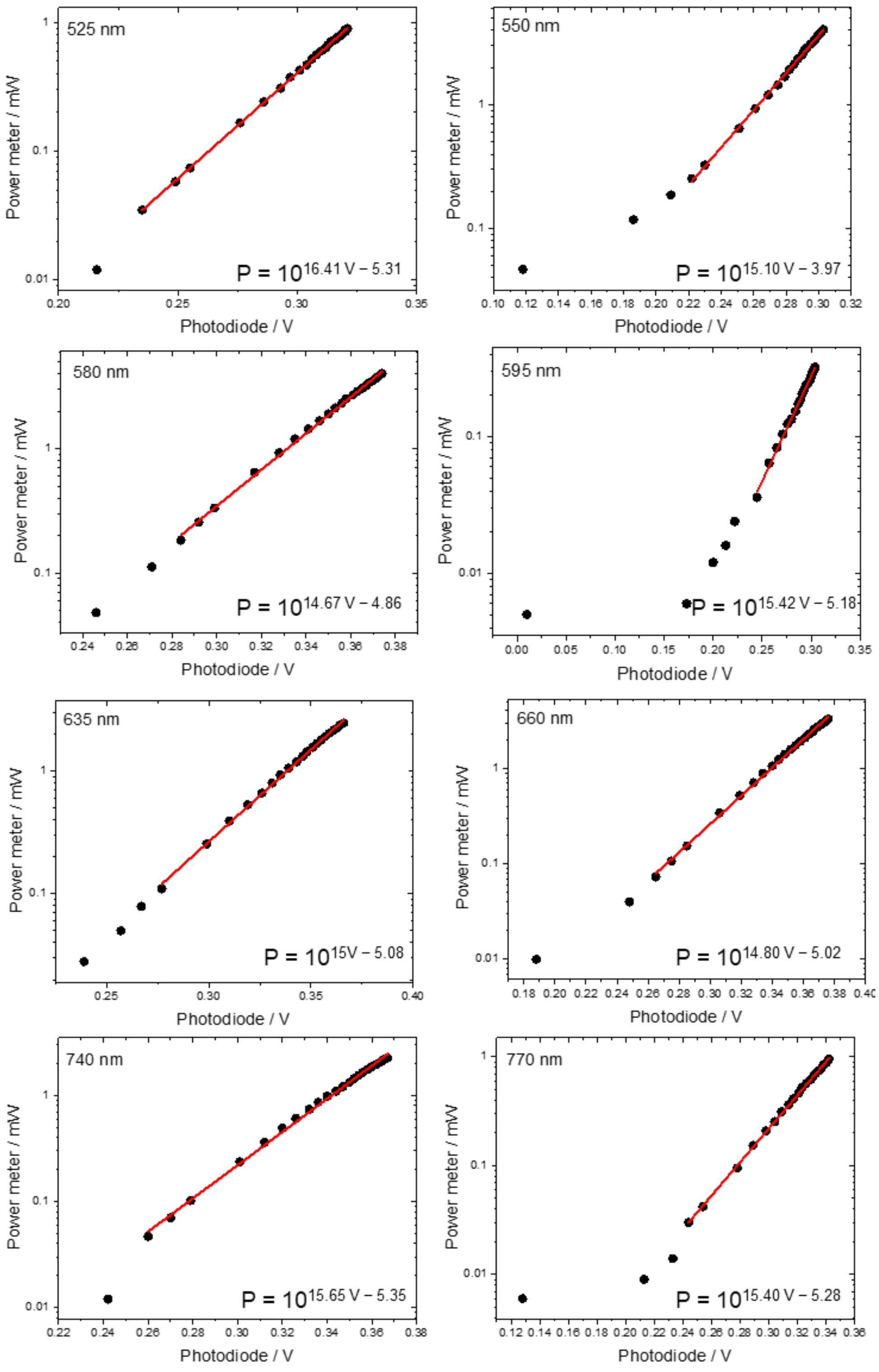

Figure S5 Calibration curves used to determine the conversion equations between the *in-situ* photodiode readings and the absolute power at different wavelengths (525-770 nm) for the coolLED system.

Table S1 Values for the slope, m, and intercept, c, obtained from the straight-line sections of the calibration plots to obtain the power delivered to the sample from the photodiode reading. Errors given are from the standard error in the slope and intercept.

System	Wavelength (nm)	Slope, m / log(mW) V^{-1}	Intercept, c / log(mW)
Prizmatix	365	16.12 ± 0.07	-4.13 ± 0.02
coolLED	365	16.80 ± 0.08	-4.18 ± 0.02
	385	16.22 ± 0.07	-4.14 ± 0.02
	405	16.42 ± 0.04	-4.23 ± 0.01
	435	16.28 ± 0.06	-4.58 ± 0.02
	460	16.05 ± 0.10	-4.64 ± 0.03
	470	17.18 ± 0.10	-5.28 ± 0.03
	490	16.57 ± 0.07	-4.99 ± 0.02
	500	16.08 ± 0.08	-5.03 ± 0.02
	525	16.41 ± 0.06	-5.31 ± 0.02
	550	15.10 ± 0.10	-3.97 ± 0.03
	580	14.67 ± 0.14	-4.86 ± 0.05
	595	15.42 ± 0.22	-5.18 ± 0.06
	635	15.00 ± 0.15	-5.08 ± 0.05
	660	14.80 ± 0.13	-5.02 ± 0.04
	740	15.65 ± 0.17	-5.35 ± 0.06
	770	15.40 ± 0.09	-5.28 ± 0.03

From the values of the slope (m) and intercept (c) obtained from the straight-line sections of the calibration curves, the following equations can be used to obtain the power, P, and error in the power, ΔP, at the sample position for a given wavelength, using the photodiode reading (x).

$$\text{Power, } P = 10^{\mathrm{m}x+\mathrm{c}} \quad \text{Eq. S1}$$

$$\Delta P = P \times 10^{\frac{\sqrt{(\Delta \mathrm{m}\, x)^2 + (\Delta \mathrm{c})^2}}{\mathrm{m}x+\mathrm{c}}} \quad \text{Eq. S2}$$

To obtain a value for the irradiance on the sample, the total power can be divided by the beam area ($50.9\ \mathrm{mm}^2$).

S4. Sample environment

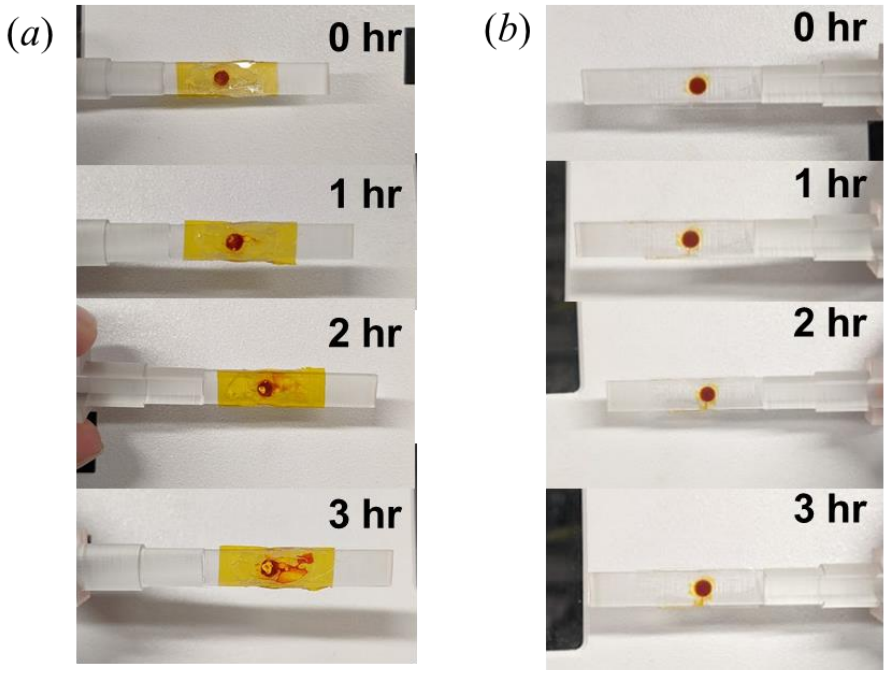

Figure S6 Light-responsive lyotropic liquid crystals in **a)** mica-Kapton and **b)** UV tape sample environments. On exposure to UV over the course of 3 hours, the sample is drawn out of the mica-Kapton holder. In contrast, the UV tape provides a better seal for the sample.

S5. Fitting to SAXS data

Table S2 Fitted parameters from SAXS data for AzoTAB (50 mM in water) after 0 and 80 minutes of UV irradiation. The 0 min sample was fitted to a core-shell ellipsoidal cylinder model and the 80 min sample was fitted to a core-shell ellipsoid model where: R_p is the polar radius and R_{eq} is the equatorial radius for the ellipsoid, t is the shell thickness, L is the cylinder length, SLD is the scattering length density for the core and shell of the micelles, Z is the fitted micelle charge, η is the micelle volume fraction and X^2 denotes the goodness of fit for the model.

UV / min	R_p / Å	R_{eq} / Å	t / Å	L / Å	SLD		Z	η	X^2
					Core	Shell			
0	31.39 ± 0.05	11.46 ± 0.04	13.28 ± 0.02	136.0 ± 0.2	5.1×10^{-6}	1.84×10^{-5}	16.9	0.08	3.6
80	13.25 ± 0.14	19.32 ± 0.20	10.25 ± 0.18		6.0×10^{-6}	1.36×10^{-5}	11.1	0.09	0.9
